# Pre-treatment loss to follow-up and treatment delay among bacteriologically-confirmed tuberculosis patients diagnosed in Mandalay Region, Myanmar

**DOI:** 10.1186/s41182-019-0154-9

**Published:** 2019-05-02

**Authors:** Ko Ko Htwe, Nang Thu Thu Kyaw, Ajay M. V. Kumar, Khine Wut Yee Kyaw, Myo Minn Oo, Thandar Thwin, Saw Saw, Si Thu Aung

**Affiliations:** 1 National TB Programme, Central-Mandalay Branch, Department of Public Health, Ministry of Health and Sports, Patheingyi Township, Mandalay Region Myanmar; 2 International Union Against Tuberculosis and Lung Disease, Mandalay, Myanmar; 3 Department of Medical Research, Yangon, Myanmar; 4 National TB Programme, Department of Public Health, Ministry of Health and Sports, Nay Pyi Taw, Myanmar

**Keywords:** Tuberculosis, Myanmar, SORT IT, Operational research, Laboratory register, Recording

## Abstract

**Background:**

Pre-treatment loss to follow-up (PTLFU) among tuberculosis (TB) patients is a global public health problem, because such patients are highly infectious and experience high mortality. There is no published evidence on this issue from Myanmar.

**Objective:**

To determine PTLFU and treatment delays (> 7 days duration between the date of diagnosis and starting anti-TB treatment) and their associated demographic, clinical, and health system-related factors among bacteriologically confirmed (sputum smear-positive and/or Xpert-positive) TB patients diagnosed in public health facilities of the Mandalay Region between January and June 2017.

**Method:**

This was a cohort study involving secondary analysis of routine programme data. Every bacteriologically confirmed TB patient in the laboratory register was tracked for at least 3 months in the treatment register. Patients neither found in the treatment register nor referred out for treatment were considered PTLFU.

**Results:**

Of the 1365 bacteriologically confirmed patients diagnosed, 1051 (77%) started on anti-TB treatment, 200 (15.6%) were referred for treatment to health facilities outside the study area, and 114 (8.4%, 95% CI 7.0%–9.9%) did not initiate anti-TB treatment (PTLFU). PTLFU was significantly higher in those with TB/HIV co-infected (18%), sputum smear-negative but Xpert MTB-positive patients (31%), and patients diagnosed at a moderate- or high-volume facility (> 50 patients tested form TB during the study period) (~ 10%). Of the 940 patients with dates recorded, 46 (5%) had a treatment delay of more than 7 days. Patients aged 45–64 years had higher risk of treatment delay compared to those aged 15–44 years. About 97% of records did not have a phone number recorded.

**Conclusion:**

PTLFU and treatment delay were relatively low in the Mandalay Region. While this is reassuring, urgent steps must be taken to address those that are lost, which includes improving documentation of phone numbers to improve ‘trackability’, instituting proactive measures to trace patients lost in the care pathway, and introducing an indicator in the national tuberculosis programme (NTP) monthly report to monitor and review PTLFU. Patient subgroups with higher PTLFU should receive priority attention.

## Background

Myanmar is one of the 30 high tuberculosis (TB) burden countries ranking 11th globally and 4th in the Southeast Asia region after India, Bangladesh, and Indonesia. In 2016, an estimated 191,000 people developed TB and 30,000 of them died in Myanmar [1].

Early detection and rapid initiation of treatment of all TB patients are necessary to reduce mortality, morbidity, and disease transmission in the community as well as in health care settings (nosocomial transmission). In 2016, the TB case detection rate in Myanmar was 72% [2]. This implies that 28% of TB patients were either not diagnosed and treated or not notified to the national TB programme. Identifying these ‘missing’ patients and linking them to appropriate care is important for Myanmar to end TB as envisaged by the END-TB strategy of the World Health Organization and United Nations’ Sustainable Development Goals [3, 4].

One of the possible reasons for ‘missing’ patients is pre-treatment loss to follow-up (PTLFU), which is defined as the proportion of diagnosed TB patients not registered on treatment. Studies on the TB care cascade show that PTLFU exceeds the combined rates of loss to follow-up (LFU), death, and failure after initiation of treatment [5]. Several studies conducted across the globe have shown that the PTLFU among TB patients varies from 4 to 38%, 18% (95% confidence intervals (CI) 13–22) in Africa and 13% (95% CI 10–15) in Asia [6]. Such patients, when untreated, are likely to transmit the disease to others and eventually die due to the disease [7–9]. Unfortunately, national TB programmes (NTPs) in most countries do not routinely report PTLFU and monitor this indicator.

The other related issue is the delay in initiating TB treatment (defined as > 7 days duration between the date of diagnosis and starting anti-TB treatment), as reported by many studies from high TB burden countries [10–15].

However, in Myanmar, there is no published evidence on the magnitude of PTLFU and the delays in treatment initiation among TB patients. In this study, we aimed to determine PTLFU and delays in starting treatment and their associated demographic, clinical, and health-system related factors among bacteriologically confirmed TB patients (sputum smear-positive and/or Xpert MTB-positive) diagnosed in public health facilities in Mandalay region between January and June 2017. We excluded patients diagnosed in private clinics and hospitals in this study due to the lack of systematic recording and reporting to NTP.

## Methods

### Design

This was a cohort study.

### Study setting

Mandalay is one of the 15 regions/states of Myanmar with a population of 5.1 million [16]. Mandalay is located in central Myanmar and administratively divided into 28 townships.

The NTP carries the overall responsibility for TB control in the Mandalay Region. TB control activities are implemented at township level as part of integrated primary health care provision by township TB teams located at township public health centres, consisting of medical officer, laboratory technician, staff nurse, and junior TB worker, under the guidance, supervision, and monitoring of the district, state/region, and central units of NTP. The basic health staffs are trained for TB control at the grass root level. While most TB patients are managed in the public sector under NTP, a small proportion is diagnosed and treated in private clinics and hospitals, especially in urban areas, which are not under the supervision of NTP in this setting.

### Diagnosis of TB

**Fig. 1:**
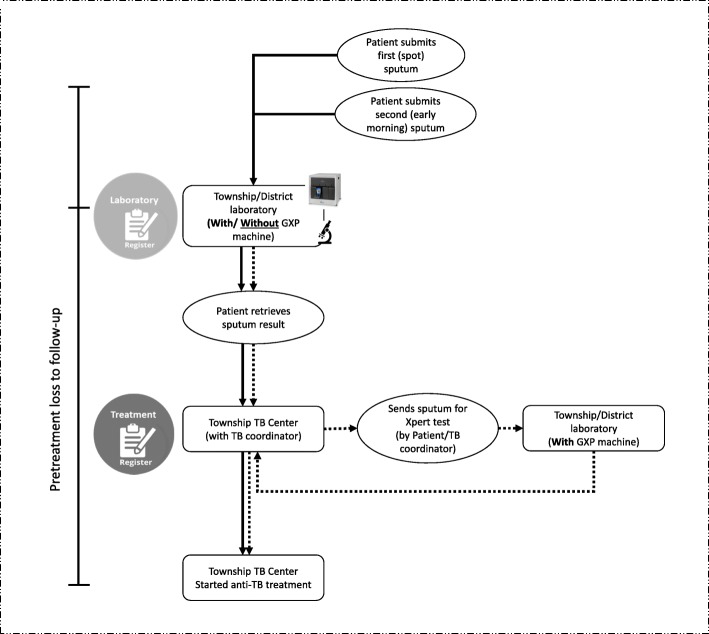
The process of diagnosis and linkage to care for bacteriologically confirmed tuberculosis patients in Myanmar between January and June 2017. TB, tuberculosis; GXP, Gene Xpert machine; Xpert test, Xpert® MTB/RIF assay; solid line, the process if the sputum smear was tested at facility with GXP machine; dotted line, the process if the sputum smear was tested at facility without GXP machine

The process of diagnosis and linkage to care for bacteriologically confirmed tuberculosis patients in Myanmar is shown in Fig. 1. Presumptive TB patients, defined as those with cough > 2 weeks, fever, night sweats, weight loss, or enlarged lymph nodes, identified at the health facilities or in community-based active case finding projects are referred to the nearest township TB laboratory. In some instances, sputum specimens of presumptive TB patients are collected and transported to the laboratory. The diagnosis of TB is primarily based on direct sputum smear microscopy. Two sputum samples (one spot and one early morning specimen) are examined to diagnose pulmonary TB by using light microscopy with Ziehl-Neelsen stain or florescence microscopy using auramine stain.

Xpert® MTB/RIF assay (Xpert test) is used primarily for the diagnosis of rifampicin resistance, except among people living with HIV (PLHIV) where it is used for the diagnosis of TB along with sputum microscopy. So, all presumptive TB patients, except PLHIV, are first tested using sputum microscopy and, if found smear-positive for acid-fast bacilli, are started on the first-line TB treatment and further tested for rifampicin resistance by Xpert test as per NTP guidelines. If the Xpert test is available in the same health facility, an additional specimen is collected and tested. In health facilities without Xpert test, smear-positive TB patients are referred to the nearest laboratory for Xpert test. Sputum smear and Xpert test results are routinely recorded in the laboratory register. Based on the results of the Xpert test (rifampicin resistant or sensitive), patients are either continued on first-line treatment or changed to second-line treatment. Among presumptive TB patients with HIV, both sputum smear microscopy and Xpert testing are done simultaneously, and based on the results, the appropriate treatment is initiated.

In the Mandalay Region, there are a total of 62 microscopy centres. Of these, eight laboratories also perform Xpert® MTB/RIF assay. In 2016, a total of 4186 bacteriologically confirmed patients were registered for treatment in the Mandalay Region [17].

### Treatment for TB

TB patients are registered in the TB treatment register and started on treatment by the township TB coordinator at township TB centres. Patients diagnosed as out-patients in general, station, and TB hospitals are referred to the respective township TB centre for treatment initiation and registration. TB patients diagnosed among in-patients are treated while hospitalised and then referred to the respective township for the continuation of treatment. TB patients diagnosed from community-based active case finding activities are linked to treatment by the community volunteers. TB diagnosis and treatment is provided free of cost to all the patients.

### Study population

All bacteriologically confirmed TB patients diagnosed at 21 township public health centres in the Mandalay Region of Myanmar between January and June 2017 were included. These 21 facilities were conveniently selected out of 28 township public health centres based on availability and access to programme data. We excluded private clinics and hospitals, clinics run by non-governmental organisations, and patients diagnosed with rifampicin resistance diagnosed by Xpert MTB/RIF test as these patients have different bacteriological and clinical profiles and have already been studied before [18].

### Data variables, sources, and management

Data collection and entry was done between December 2017 and April 2018. To facilitate efficient tracking, we digitised the TB laboratory and treatment registers. Digitisation was done in a quality-assured (double entry validation) manner using EpiData software (v3.1, EpiData Association, Odense, Denmark) by trained data entry operators.

#### Digitisation of laboratory register

The study cohort was constituted by digitising the TB laboratory registers for the period January–June 2017. The variables included name, age, sex, HIV status, previous history of TB treatment, results of sputum microscopy and Xpert MTB/RIF, name of health facility, phone number (recorded or not), date of diagnosis, and referral for treatment. It is expected that the patients are tested at multiple laboratories especially in situations where Xpert test services are not available in the same health facility. We, therefore, searched the laboratory database for duplicate patient entries and removed them.

#### Digitisation of treatment register

We then digitised the TB treatment registers for the period January–September 2017. This enabled the tracking of every diagnosed TB patient for at least 3 months, until September 30, 2017. Variables extracted from the TB register include name, age, sex, name of health facility, laboratory serial number, HIV status, and date of treatment initiation. If there was a discrepancy in variables between laboratory and treatment registers, laboratory register was considered final for all variables except HIV status. For HIV status, the information from TB register was used to update the information in the laboratory register.

#### Matching of laboratory and treatment registers

For each patient listed in the laboratory database, we searched the treatment database to assess for PTLFU. We used the VLOOKUP function in MS Excel to match the laboratory and treatment databases using the following variables: laboratory serial number, patient name, age, sex, and township. The laboratory number and patient name were the primary tracking variables, while the other variables were used for confirmation. For records where we did not find an exact match (due to variations in the spellings of patient name), we looked at the other variables and decided this was a ‘match’ only if we had a perfect match for sex and township and an approximate match for age (± 2 years).

#### Operational definitions

*Bacteriologically confirmed TB patients:* Patients whose sputum smear and/or Xpert MTB results were positive in the laboratory register.

*Started on anti-TB treatment:* Patients found in the treatment database as per the above-mentioned criteria were considered ‘Started on anti-TB treatment’.

*Referral for treatment:* Patients who were documented in the laboratory register to have been referred out of the study area for treatment were considered ‘Referral for treatment’.

*PTLFU:* Bacteriologically confirmed TB patients found in the laboratory database but were neither referred out nor found in the treatment database were considered PTLFU.

*Treatment delay:* If the duration between the date of diagnosis and treatment start was more than 7 days, it was considered ‘treatment delay’.

*Categories of health facilities:* Based on the number of sputum-positive patients diagnosed in the study period, health facilities were classified into three categories: (i) high volume (> 70 patients), (ii) moderate volume (50–70 patients), and (iii) low volume (< 50 patients). The cut-offs were chosen based on the median and 75th percentile.

### Data analysis and statistics

Data was analysed using EpiData (version 2.2.2.183) and Stata (version 12, Texas, USA) software. We used medians and interquartile ranges (IQR) or numbers and percentages to describe the patient characteristics. We assessed the associations of demographic and clinical factors with outcomes using relative risks (RR) and 95% confidence intervals (CI). Factors with *p* value < 0.2 in unadjusted analysis and age and sex were included in a multivariable model (log-binomial regression or Poisson regression with robust error estimates if convergence was not obtained in the binomial model) to determine the independent factors associated with outcomes. Level of significance was set at 5%. We adhered to the STROBE (Strengthening The Reporting of Observational studies in Epidemiology) guidelines for conduct and reporting of the study [19].

## Results

There were 1365 bacteriologically confirmed TB patients in the study. Median age of participants was 40 years (IQR 30–52 years), 1018 (75%) were male, 71 (5%) were recorded as HIV-positive, 110 (8%) had a previous history of anti-TB treatment, and 729 (53%) were diagnosed from a ‘high volume’ facility (Table 1).

**Table 1:** Demographic and clinical profile of bacteriologically-confirmed TB patients diagnosed in 21 township health centres of Mandalay Region of Myanmar between January and June 2017

Characteristics	Number (%)
Total	1365 (100)
Age (years)	
< 15	8 (0.6)
15–44	805 (59.9)
45–64	425 (31.1)
≥ 65	127 (9.3)
Gender	
Male	1018 (74.6)
Female	347 (25.4)
Phone number recorded or not	
Yes	38 (2.8)
No	1327 (97.2)
HIV status	
Positive	71 (5.2)
Negative	1060 (77.7)
Unknown	234 (17.1)
History of TB treatment	
Yes	110 (8.1)
No	864 (63.3)
Unknown	391 (28.6)
Sputum smear result	
Positive	1303 (95.5)
Negative	62 (4.5)
Xpert MTB/RIF result	
No MTB	28 (2.1)
Invalid	2 (0.2)
MTB detected	636 (46.6)
Not recorded	699 (51.2)
Site of sputum microscopy	
Low-volume facility	308 (22.6)
Moderate-volume facility	328 (24)
High-volume facility	729 (53.4)

*TB* tuberculosis, *MTB* mycobacterium tuberculosis, *RIF* rifampicin

### PTLFU

**Fig. 2:**
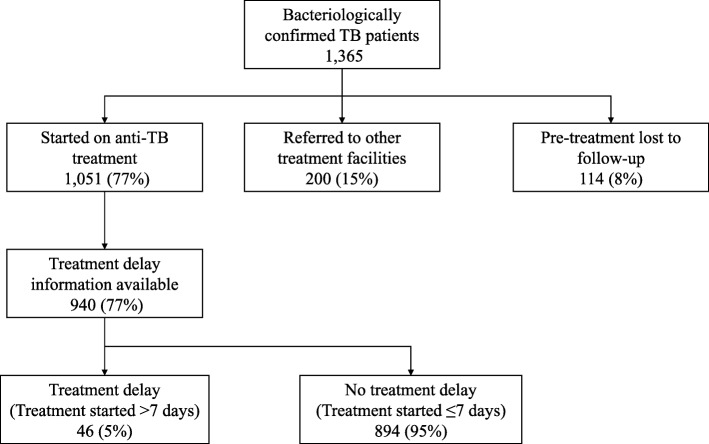
Flow chart of bacteriologically confirmed TB patients diagnosed in 21 township health centres of Mandalay Region of Myanmar between January and June 2017. TB, tuberculosis

Of the TB patients diagnosed, 1051 (77%) were started on anti-TB treatment, 200 (15.6%) were referred for treatment to health facilities outside the study area, and 114 (8.4%) did not initiate anti-TB treatment (Fig. 2). Thus, the proportion of PTLFU among the bacteriologically confirmed TB patients was 8.4% (95% CI 7.0–9.9%). If patients referred for treatment were excluded from the denominator, then PTLFU was 9.8% (95% CI 8.2–11.6%). The PTLFU was higher in some patient subgroups like HIV-positive patients (18.3%), previously treated patients (20%), and smear-negative but Xpert-positive patients (30.7%). In addition, patients diagnosed at a moderate or high-volume facility were significantly associated with higher risk of PTLFU (Table 2).

**Table 2:** Factors associated with pre-treatment loss to follow-up among bacteriologically confirmed TB patients diagnosed in 21 township health centres of the Mandalay Region of Myanmar between January and June 2017

Characteristics	Total (*n*)	PTLFU (*n* (%))	RR (95%CI)	aRR (95%CI)
Total	1365	114 (8.4)		
Age (years)				
< 15	8	2 (25.0)	3.0 (0.9–10.2)	1.3 (0.3–5.4)
15–44	805	67 (8.3)	Ref	Ref
45–64	425	38 (8.9)	1.1 (0.7–1.6)	0.9 (0.6–1.4)
≥ 65	127	7 (5.5)	0.7 (0.3–1.4)	0.6 (0.2–1.3)
Gender				
Male	1018	86 (8.5)	Ref	Ref
Female	347	28 (8.1)	0.9 (0.6–1.4)	1.0 (0.65–1.55)
Phone number				
Recorded	38	1 (2.6)	0.3 (0.0–2.2)	
Not recorded	1327	113 (8.5)	Ref	
HIV status				
Positive	71	13 (18.3)	**3.5 (2.0–6.1)**	**2.7 (1.4–5.0)**
Negative	1060	55 (5.2)	Ref	Ref
Unknown	234	46 (19.7)	**3.8 (2.6–5.5)**	**4.2 (2.7–6.4)**
History of TB treatment				
Yes	110	22 (20.0)	**2.5 (1.6–3.9)**	1.7 (0.9–2.8)
No	864	68 (7.9)	Ref	Ref
Unknown	391	24 (6.1)	0.8 (0.5-1.2)	**0.5 (0.3–0.8)**
Sputum result				
Smear positive	1303	95 (7.3)	Ref	Ref
Smear negative Xpert positive	62	19 (30.7)	**4.2 (2.8–6.4)**	**2.6 (1.4–4.6)**
Site of sputum microscopy				
Low volume	308	11 (3.6)	Ref	Ref
Moderate volume	328	27 (8.2)	**2.3 (1.2–4.6)**	**2.2 (1.1–4.4)**
High volume	729	76 (10.4)	**2.9 (1.6–5.4)**	**1.7 (0.9–3.2)**

*PTLFU* pre-treatment loss to follow-up, *TB* tuberculosis, *RR* relative risk, *aRR* adjusted relative risk (adjusted for age, gender, HIV status, history of TB treatment, sputum result, site of sputum microscopy), *Ref* reference group. Text in boldface indicates the statistical significance at *p* value < 0.001

### Treatment delay

Of the 940 patients with valid dates of diagnosis and treatment start, 46 (5%) had a treatment delay of more than 7 days. The median duration between diagnosis and treatment start was 1 day (IQR 0–2 days). Patients aged 45–64 years had a higher risk of treatment delay compared to those aged 15–44 years (Table 3).

**Table 3:** Factors associated with treatment delay among bacteriologically confirmed TB patients who received treatment in 21 township health centres of the Mandalay Region of Myanmar between January and June 2017

Variables	Total (*n*)	Treatment delay (*n* (%))	RR (95%CI)	aRR (95% CI)
Total	940	46 (4.9)		
Age (years)				
< 15	3	0 (0.0)		
15–44	536	18 (3.4)	Ref	Ref
45–64	308	21 (6.8)	**2.0 (1.1–3.8)**	**1.9 (1.0–3.6)**
≥ 65	93	7 (7.5)	2.2 (0.9–5.2)	2.0 (0.8–4.9)
Gender				
Male	701	36 (5.1)	Ref	Ref
Female	239	10 (4.2)	0.8 (0.4–1.6)	0.8 (0.4–1.8)
Phone number				
Recorded	31	2 (6.5)	0.3 (0.0–2.2)	
Not recorded	909	44 (4.8)	Ref	
HIV status				
Positive	46	4 (8.7)	2.03 (0.7–5.5)	1.86 (0.6–5.4)
Negative	864	37 (4.3)	Ref	Ref
Unknown	30	5 (16.7)	3.89 (1.6–9.2)	3.76 (1.4–9.9)
History of TB treatment				
Yes	67	6 (8.9)	2.08 (0.9–4.9)	1.89 (0.7–4.8)
No	630	27 (4.3)	Ref	Ref
Unknown	243	13(5.4)	1.24 (0.6–2.4)	1.15 (0.6–2.3)
Sputum result				
Smear positive	913	43 (4.7)	Ref	Ref
Smear negative Xpert positive	27	3 (11.1)	2.35 (0.8–7.1)	1.46 (0.4–5.1)
Not recorded	135	12(8.9)	0.73 (0.4–1.4)	
Site of sputum microscopy				
Low volume	264	10 (3.8)	Ref	Ref
Moderate volume	261	13 (4.9)	1.3 (0.6–2.9)	1.5 (0.6–3.4)
High volume	415	23 (5.5)	1.5 (0.7–3.0)	1.6 (0.7–3.4)

*TB* tuberculosis, *RR* relative risk, *aRR* adjusted relative risk (adjusted for age, gender, HIV status, history of TB treatment, sputum result, site of sputum microscopy), *Ref* reference group. Text in boldface indicates the statistical significance at *p* value < 0.001

### Completeness of records in laboratory register

Of the 1365 records evaluated, 1327 (97%) records did not have phone number recorded, 666 (49%) records did not have HIV testing information, and 699 (51%) did not have Xpert MTB/RIF test results. Of the 1327 patients who did not have a phone number in laboratory register, 926 (70%) had a phone number recorded in the treatment register. Of the 699 patients lacking Xpert results in the laboratory register, 301 (43%) had Xpert results in the treatment register.

## Discussion

This is the first study from Myanmar reporting PTLFU and treatment delay among bacteriologically confirmed patients without known rifampicin resistance. It was reassuring to find that PTLFU and treatment delays were relatively lower compared to average PTLFU rates and delays reported in African and Asian settings [6, 11, 20–22].

We believe PTLFU of 8% is an underestimate for the following reasons. First, we included patients ‘referred for treatment’ as part of the denominator. If they were excluded from the denominator, as has been the case in most of the previous studies, PTLFU increases marginally to ~ 10%. Programme experience indicates that most of the patients with documented referral start treatment. However, this needs to be formally studied.

Second, we had excluded many high-volume health facilities in the Mandalay Region from our study, due to operational difficulties of accessing data. These facilities attract patients from all over the region, and PTLFU is likely to be higher in patients diagnosed in such facilities, possibly due to long distances, patient mobility, and losses in the complicated referral process after diagnosis. While it is possible that some of the patients declared PTLFU in our study may have been started on treatment in the private health sector, we believe such instances are limited and may not have impacted our estimate of PTLFU greatly. This needs further study.

We identified several factors associated with PTLFU in our study. HIV-infected TB patients experienced a higher risk of PTLFU, probably due to higher case fatality in this group, which in turn may be due to late presentation to the health facility coupled with severe illness. This has been shown in several studies from Africa, the region most severely affected by the HIV-TB epidemic [23]. A previous qualitative study from South Africa reports stigma as one of the other reasons for PTLFU among HIV-TB patients [24].

Previously treated TB patients had a higher risk of PTLFU compared to new patients, as has been reported by a recent study in India [21]. This may again be due to severe illness at presentation and higher case fatality in this group.

Patients who were sputum smear-negative but Xpert-positive had a higher risk of PTLFU. This is a novel risk factor identified, but not reported in previous studies. Although we do not know the exact reasons, we speculate that this may be related to the profile of patients and access to Xpert testing. In Myanmar, sputum smear-negative presumptive TB patients are offered Xpert testing only if they have accompanying HIV or diabetes mellitus or if they are a contact of multidrug-resistant TB patients or on request of the specialist physicians in selected, severely ill, sputum-negative patients with high index of suspicion of TB. This needs further exploration in future studies.

Another risk factor identified in our study was related to the volume of patients diagnosed in health facilities. Barring a recent study from India, no other study has examined this aspect [21]. Similar to the study from India, we also found that patients diagnosed in high-volume and moderate-volume facilities had a higher risk of PTLFU compared to low-volume health facilities [21]. We speculate that this may be due to two reasons: (i) patients diagnosed in low-volume facilities are more likely to be residing close to the facilities whereas high-volume facilities (generally referral facilities) are likely to attract patients from distant areas and (ii) patients in low-volume facilities may have had more favourable provider-patient interactions compared to high-volume facilities, where the staff are often overburdened and the time spent per patient is likely to be less.

The documentation of phone numbers was poor in the laboratory register, similar to other settings [25]. This is an index of ‘trackability’ of TB patients, and previous studies have shown that this is an important factor associated with PTLFU [21]. We believe most of the TB patients in Myanmar have access to mobile phones, and documenting it in the laboratory register will help in actively tracking ‘lost’ patients.

Only 1 in 20 patients had a treatment delay of more than 7 days in our study, as compared to 42% of patients experiencing a delay in a study from India [11]. The median delay in our study was just 1 day, compared to 2 days in a study from Ethiopia [26]. We found TB patients aged 45 years and above had a higher risk of delay. So, older patients should be a focus of our interventions aiming to reduce the delays. There may be other factors associated with treatment delay which we did not collect data on. Delay has been associated with both patient-related factors (male, low income, severity of disease, unemployment, poor knowledge of TB) and health system-related factors (lack of qualified providers, long travel times and distances to a health facility) [10–15].

There were a couple of methodological strengths in our study. First, we employed a systematic method to digitise the laboratory and treatment registers and matched the two databases using a combination of electronic tracking, backed up by manual checking for every patient. This minimised the misclassification of PTLFU. Second, we performed a cohort analysis, thus providing a more accurate estimate of PTLFU, compared to that obtained by comparison of aggregate data routinely reported by NTP.

The study also had some limitations. As discussed above, we may have underestimated the magnitude of PTLFU. Also, we need to exercise caution before generalising the results to other regions of the country. Given the differences in geography, ethnicity, and access to health care in different regions of Myanmar, we feel PTLFU and delays may differ. Similar research studies should be conducted in other regions and states for obtaining a national picture. Third, we did not know what happened to patients who were PTLFU. We did not actively track the ‘lost’ patients. Such efforts would have provided insights into the reasons for PTLFU and the potential yield of an active patient tracking system. Future operational research should focus on this issue using a combination of quantitative and qualitative research methods. Finally, there were missing data with reference to some key variables which might have affected the internal validity of our analysis.

There are several programme implications from this study. First, urgent measures are required to track patients not initiated on treatment. Such patients not only have high mortality and morbidity, but are also the source of continued disease transmission in the community and hospitals. Previous studies have shown that having a dedicated team to track patients is helpful in reducing the magnitude of PTLFU [6, 16]. In the absence of such a team, the laboratory technician and the TB coordinator should meet periodically and exchange information. The patient’s phone number and addresses should be properly documented in the laboratory register and used to improve the tracking of patients.

Second, NTP should introduce an indicator in the monthly or quarterly report to monitor PTLFU, which has suffered the neglect of programmes and policymakers globally, since it was first described five decades ago [27]. As the adage goes, ‘What gets monitored, gets done’. So, introducing the monitoring indicator and reviewing it periodically will sensitise the programme staff to pay attention to this neglected issue. The information on TB treatment initiation along with TB number should be recorded in the laboratory register, as recommended by the NTP in Myanmar. This enables easy cohort analysis compared to the complicated matching of records undertaken in this study (based on name, age, and other variables which are not often accurately documented). We also recommend documenting a government-issued unique identifier in both laboratory and treatment registers which helps in efficient tracking.

Third, to be able to track the whole cascade of TB diagnosis and treatment, the use of presumptive TB registers or cough registers at the township health centres should be strengthened. Using comprehensive electronic recording systems may be another option to improve the recording and tracking of patients, but this may require a significant investment of resources [25].

Fourth, patient cohorts for assessing treatment outcomes should be constituted from ‘laboratory registers (all diagnosed patients)’ and not ‘treatment registers (all treated patients)’ so that PTLFU is accounted for when assessing patient outcomes [28]. There are two consequences of doing this: an increase in case detection rates and a decrease in treatment success rates. If we extrapolate using the PTLFU rates found in our study to Myanmar, the treatment success rate may decrease from 87 to 80%. But this step transparently and truthfully acknowledges the problem, which is often the first step in addressing it.

Finally, by not accounting for PTLFU in treatment outcomes, NTPs across the globe are deceiving themselves by reporting inflated treatment success rates. We recommend the World Health Organization to introduce a dedicated indicator in the global TB report and mandate this information from all countries. This is also essential to measure the third ‘90’ (successfully treating 90% of all ‘diagnosed’ TB patients) of the 90-90-90 targets set by the Stop TB Partnership to measure the progress of countries towards ending TB [29].

## Conclusion

In conclusion, we found a relatively low level of PTLFU and treatment delay among TB patients in the Mandalay Region of Myanmar. While this is reassuring, urgent steps must be taken to address it which includes improving the documentation of phone numbers to improve ‘trackability’, instituting proactive measures to trace patients lost in the care pathway, and introducing an indicator in the NTP monthly report to monitor and review PTLFU. Patient subgroups with higher PTLFU should receive priority attention.

## Abbreviations

CI: Confidence intervals
HIV: Human immunodeficiency virus
IQR: Interquartile range
LFU: Loss to follow-up
NTP: National TB programme
PLHIV: People living with HIV
PTLFU: Pre-treatment loss to follow-up
RR: Relative risks
STROBE: Strengthening The Reporting of Observational studies in Epidemiology
TB: Tuberculosis
